# Systematic Comparison of C3 and C4 Plants Based on Metabolic Network Analysis

**DOI:** 10.1186/1752-0509-6-S2-S9

**Published:** 2012-12-12

**Authors:** Chuanli Wang, Longyun Guo, Yixue Li, Zhuo Wang

**Affiliations:** 1 Department of Bioinformatics and Biostatistics, Shanghai Jiao Tong University, 800 Dongchuan Road, Shanghai, 200240, China; 2 Key Laboratory of Systems Biology, Shanghai Institutes of Biological Sciences, Chinese Academy of Sciences, 320 Yueyang Road,Shanghai, 200031, China

## Abstract

**Background:**

The C4 photosynthetic cycle supercharges photosynthesis by concentrating CO_2 _around ribulose-1,5-bisphosphate carboxylase and significantly reduces the oxygenation reaction. Therefore engineering C4 feature into C3 plants has been suggested as a feasible way to increase photosynthesis and yield of C3 plants, such as rice, wheat, and potato. To identify the possible transition from C3 to C4 plants, the systematic comparison of C3 and C4 metabolism is necessary.

**Results:**

We compared C3 and C4 metabolic networks using the improved constraint-based models for Arabidopsis and maize. By graph theory, we found the C3 network exhibit more dense topology structure than C4. The simulation of enzyme knockouts demonstrated that both C3 and C4 networks are very robust, especially when optimizing CO_2 _fixation. Moreover, C4 plant has better robustness no matter the objective function is biomass synthesis or CO_2 _fixation. In addition, all the essential reactions in C3 network are also essential for C4, while there are some other reactions specifically essential for C4, which validated that the basic metabolism of C4 plant is similar to C3, but C4 is more complex. We also identified more correlated reaction sets in C4, and demonstrated C4 plants have better modularity with complex mechanism coordinates the reactions and pathways than that of C3 plants. We also found the increase of both biomass production and CO_2 _fixation with light intensity and CO_2 _concentration in C4 is faster than that in C3, which reflected more efficient use of light and CO_2 _in C4 plant. Finally, we explored the contribution of different C4 subtypes to biomass production by setting specific constraints.

**Conclusions:**

All results are consistent with the actual situation, which indicate that Flux Balance Analysis is a powerful method to study plant metabolism at systems level. We demonstrated that in contrast to C3, C4 plants have less dense topology, higher robustness, better modularity, and higher CO_2 _and radiation use efficiency. In addition, preliminary analysis indicated that the rate of CO2 fixation and biomass production in PCK subtype are superior to NADP-ME and NAD-ME subtypes under enough supply of water and nitrogen.

## Background

**Figure 1:**
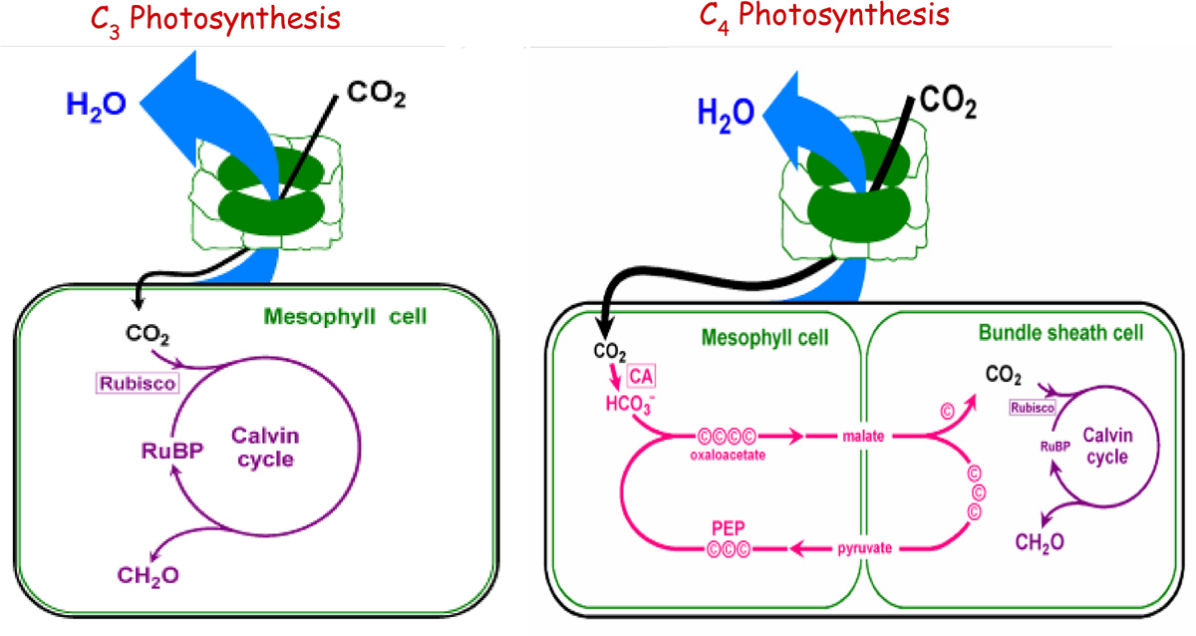
**A schematic diagram of C3 and C4 photosynthesis**.

C4 plants such as maize, sorghum, and sugarcane, approximately have 50% higher photosynthesis efficiency than those of C3 plants such as rice, wheat, and potato [1]. This is because the different mechanism of carbon fixation by the two types of photosynthesis, as illustrated in Figure 1. C3 photosynthesis only uses the Calvin cycle for fixing CO_2 _catalyzed by ribulose-1,5-bisphosphate carboxylase (Rubisco), which takes place inside of the chloroplast in mesophyll cell. For C4 plants such as maize (NADP-ME subtype), photosynthetic activities are partitioned between mesophyll and bundle sheath cells that are anatomically and biochemically distinct. The initial carbon fixation is catalyzed by phosphoenolpyruvate carboxylase (PEPC) forming oxaloacetate (OAA) from CO_2 _and phosphoenolpyruvate (PEP). OAA is metabolized into malate, and then diffuses into the BS cell where it is decarboxylated to provide increased concentration of CO_2 _around Rubisco. Finally, the initial substrate of the C4 cycle, PEP, is regenerated in mesophyll cell by pyruvate orthophosphate dikinase (PPDK) [1]. The CO_2 _concentration mechanism suppresses the oxygenation reaction by Rubisco and the subsequent energy-wasteful photorespiratory pathway, resulting in increased photosynthetic yield and more efficient use of water and nitrogen comparing to C3 plants [2]. Therefore genetic engineering of C4 features into C3 plants such as rice (Oryza sativa) has the potential to increase crop productivity [3-5]. However, attempts to use these tools to engineer plant metabolism have met with limited success due to the complexity of plant metabolism. Genetic manipulations rarely cause the predicted effects, and new rate-limiting steps prevent the accumulation of some desired compounds [6,7].

In a bid to improve our understanding of plant metabolism and thereby the success rate of plant metabolic engineering, a systems-based framework to study plant metabolism is needed [7,8]. Systems biology involves an iterative process of experimentation, data integration, modeling, and generation of hypotheses [9,10]. With the recent advancement of genome sequencing, several plants have complete genomic sequence and annotation, including Arabidopsis (Arabidopsis thaliana) [11], rice (Oryza sativa), sorghum (Sorghum bicolor) [12], and maize (Zea mays), which make it possible to reconstruct the genome-scale metabolic network of plants. Constraint-based model, also called Flux Balance Analysis (FBA), is a useful method to analyze large-scale metabolic network without requiring detail kinetic parameters. In FBA, flux states are predicted which are optimal with regard to an assumed cellular objective such as maximizing biomass yield [13-16]. For microbial organisms, FBA has been successful in predicting *in vivo *maximal growth rate, substrate preference and the requirement for particular biochemical reactions for cellular growth [17]. For plants, highly compartmentalized stoichiometric models have been developed for barley seeds [18] and Chlamydomonas [14], especially several models have been reported for Arabidopsis [19-22]. In addition, the analysis of metabolic network for photosynthetic bacteria has also been conducted, such as Synechocystis [23] and purple nonsulfur bacteria [24].

**Figure 2:**
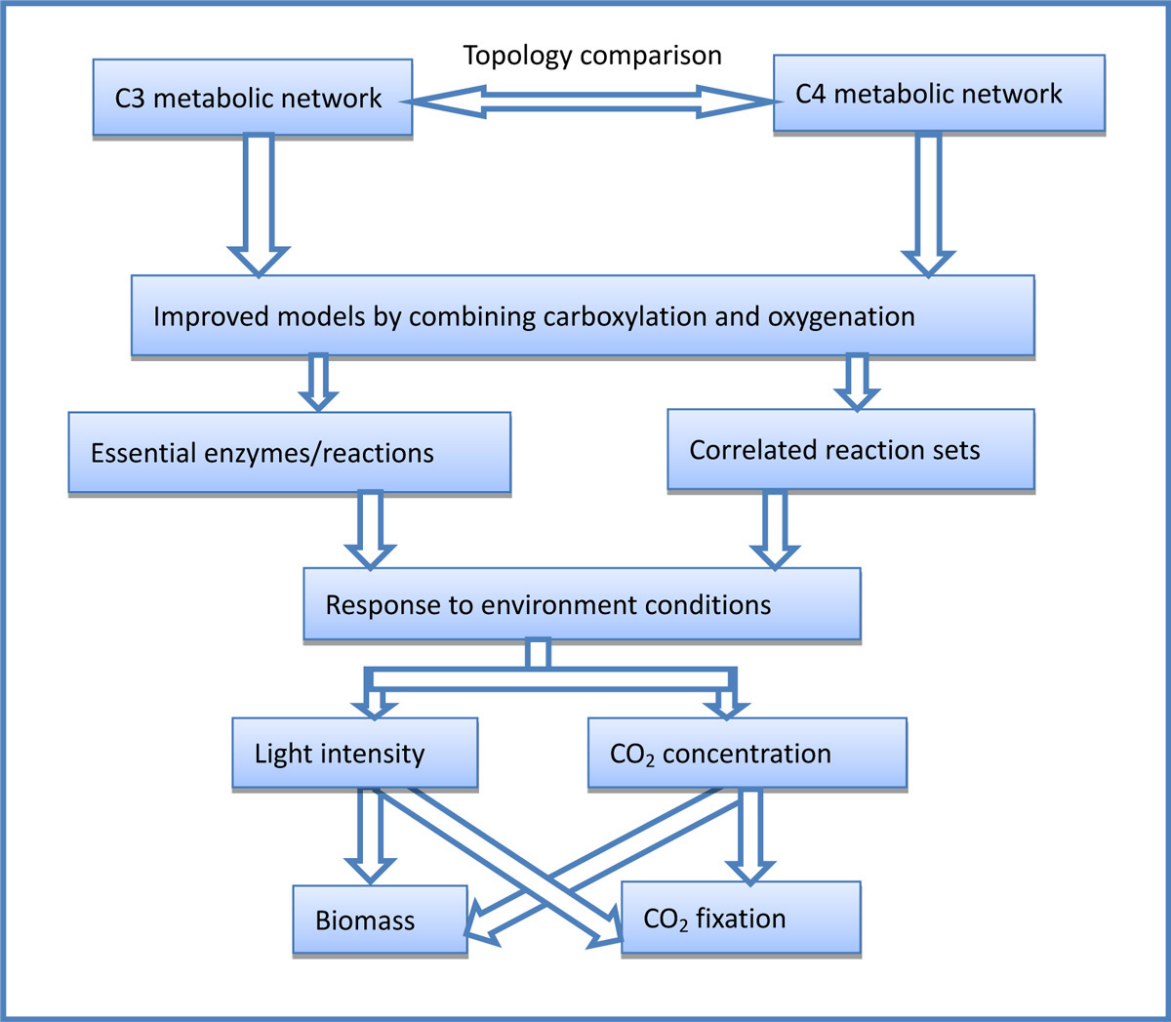
**System flow of the comparison between C3 and C4 metabolic networks**.

The genome scale metabolism models of C3 plant Arabidopsis [19] and C4 plant [25] have been constructed, but no comparative analysis between them. In this study, we improved the two models, AraGEM and C4GEM, by setting ratio of carboxylation and oxygenation by Rubisco, and compared the differences of network structure and metabolic flux to elucidate the evolutionary significance. We explored the effects of enzyme knockouts on photosynthesis and biomass synthesis, and compared the contribution of different C4 subtypes to biomass production. In addition, we revealed the different response to environment conditions in C3 and C4 plants. The system flow of our analysis is shown in Figure 2. This study will shed light on the metabolism changes from C3 to C4 at systems level, which is important for feasible engineering of C3 to C4 plants.

## Results and Discussion

### Topological characteristics of C3 and C4 metabolic networks

The metabolism model of Arabidopsis AraGEM includes 1498 unique reactions, 1765 metabolites, 83 inter-organelle transporters, and 18 inter-cellular transporters [19]. For the metabolism model of C4 plants C4GEM, there are 2377 reactions, 2886 metabolites, 177 inter-organelle transporters, and 23 external transporters [25]. The topological properties of AraGEM and C4GEM models were analyzed using pajek [26], where reactions are represented as nodes and metabolites as edges. Some important topological parameters such as average degree, betweenness centrality, average clustering coefficient and distance were compared between these two models, as shown in Table 1.The results demonstrated that the AraGEM has a more dense structure than C4GEM, because C3 plant is single-cell, while C4 plant consists of mesophyll cell and bundle sheath cell, the connections between two-cells are not as close as single-cell. Then we extracted the primary metabolism from C3 and C4 networks, including Calvin cycle, photorespiration, TCA cycle, nitrogen metabolism, sucrose and starch metabolism, and some major amino acid metabolism pathways. Using NET-SYNTHESIS [27], we calculated the redundancy of primary metabolic network of C3 and C4, which is 0.7175 and 0.7606 respectively. It means C4 network is more redundant so that C4 plant could be more robust to gene mutation or environment changes.

**Table 1:** Topological properties of AraGEM and C4GEM

model	Average Degree	Degree Centralization	Average Clustering Coefficient	Betweenness Centralization	Average distance	Maximum distance	Redundancy of primary network
C3	91	0.24016	0.37978	0.04336	2.75825	11	0.7175
C4	56	0.11384	0.40274	0.15158	3.58215	14	0.7606

### Improved models by setting the ratio of carboxylation and oxygenation by Rubisco

Rubisco enzyme (EC: 4.1.1.39) catalyzed two different reactions with CO_2 _and O_2 _respectively in photosynthesis and photorespiration:

(1) $$\text{RuBP}+\text{C}{\text{O}}_{\text{2}}+{\text{H}}_{2}\text{O }->2\text{PGA}$$

(2) $$\text{RuBP}+{\text{O}}_{\text{2}}->\text{PGA}+\text{PGCA}$$

There is constant ratio between rate of carboxylation and oxygenation under specific partial pressure of CO_2 _and O_2 _in environment [28]. Therefore, it is hard to accurately simulate the flux change under different CO_2 _concentration without constraints on rate of the two reactions by Rubisco, which is just the limitation of AraGEM and C4GEM. Here we improved the two models by combining the two reactions into one reaction:

(3) $$\left(r+1\right)\text{RuBP}+r\;\text{C}{\text{O}}_{2}+r\;{\text{H}}_{2}\text{O}+{\text{O}}_{2}\text{ - > }\left(2r+1\right)\text{PGA}+\text{PGCA}$$

The ratio *r *between carboxylation and oxygenation under different CO_2 _concentration in C3 and C4 model is shown in Table 2. The detail calculation of *r *is in the Methods section.

**Table 2:** The ratio r between carboxylation and oxygenation under different CO_2 _concentration in C3 and C4 model

CO_2 _(μbar) in the air	*r *in C3	*r *in C4
100	1.139	22.2282
380	4.33	70.7281
550	6.26	85.9654
800	9.11	87.1062
1000	11.39	88.0189

In addition, our motivation was to compare the differences between C3 and C4 photosynthesis mechanism and their responses under different environments, therefore we set the objective function as maximization of CO_2 _fixation and biomass synthesis. Since in previous AraGEM and C4GEM, the objective was to minimize the use of light energy while achieving a specified growth rate, we need to reset some flux constraints according to biochemistry knowledge. For example, the CO_2 _leakage was blocked from bundle sheath to mesophyll cell with zero flux in C4GEM, which was not consistent with actual situation; here we adjusted the upper bound of this reaction to permit the leakage of CO_2_. In addition, because starch is not synthesized in mesophyll cell of C4 plants, the biomass components of C4GEM were also reset. The lower and upper bounds of flux in TCA cycle were adjusted as -50 and 50, to restrict flux of respiration in mitochondria. The detail of modified constraints in our improved models can be got from the Additional File.

### The effects of knock-out enzymes on metabolic flux

Based on the improved C3 and C4 metabolic networks, we compared the optimal flux of biomass synthesis and CO_2 _fixation using FBA. When biomass synthesis is the objective function, the maximal flux of biomass is 3.661 and 4.625 mmol·gDW^-1^·hr^-1 ^respectively in C3 and C4 networks. Similarly, when optimizing CO_2 _fixation, the maximal flux is 200.95 mmol·gDW^-1^·hr^-1 ^in C3 network and 387.619 mmol·gDW^-1^·hr^-1 ^in C4 network. It demonstrated that C4 network exhibited both higher fluxes of biomass and CO_2 _fixation than C3 network, which was consistent with the actual tendency. We concluded that the two genome-scale metabolic networks could explain actual situations and be compared for understanding the similarities and differences of C3 and C4 plants.

Next, we evaluated the effects of enzyme knockouts on flux of CO_2 _fixation and biomass. When one enzyme was knockout, its corresponding reactions would be deleted, which resulted in changes of the optimal flux of biomass or CO_2 _fixation. The objective results from the simulation were classified as unchanged objective (ratio = 1), reduced objective (ratio ∈ (0, 1)) and no objective (ratio = 0). The effects of single reaction deletion on maximal flux of biomass in C3 and C4 network are shown in Table 3. More than 85% reactions have no effects on the maximal biomass of C3 and C4 network when being knocked-out, so we concluded that the two networks have amazing robustness. Almost 10% of the reactions would result in zero biomass in C3 and C4 networks, which include some important transporters. The single deletion of important reactions or enzymes such as phosphoribulokinase (PRK, EC: 2.7.1.19) and light reactions can result in no biomass, which is consistent with the real characteristics of plants [29].

**Table 3:** The effects of knockout reactions on maximal flux of biomass

Ratio of objective flux	C3 reactions		C4 reactions	
	
	Number	Percentage	Number	Percentage
Ratio ≈0	169	10.58%	236	9.16%
0<Ratio<0.90	14	0.88%	6	0.23%
0.90<Ratio<1	37	2.32%	78	3.03%
Ratio = 1	1378	86.23%	2256	87.58%

The effects of single reaction deletion on C3 and C4 networks when objective function is CO_2 _fixation are shown in Table 4 which is similar with Table 3. More than 96% reactions have no influence on the maximal flux of CO_2 _fixation when being deleted in C3 and C4 networks. We concluded that more reactions have no influence on the maximal flux of CO_2 _fixation than biomass. Since biomass synthesis includes many components which deal with more than one reaction, their deletion will affect the flux of biomass synthesis. In addition, it is obvious that C4 plants exhibit much better robustness than C3 plants, since higher percentage of enzyme knockouts result no change on the objective flux and lower percentage result in zero flux. Moreover, we found all the essential reactions in C3 network are also essential for C4, while there are some other reactions specifically essential for C4. This result proved that the basic metabolism of C4 plants was similar to C3, but C4 became more complex during long period of evolution.

**Table 4:** The effects of knockout reactions on maximal flux of CO_2 _fixation

Ratio of objective flux	C3 reactions		TC4 reactions	
	
	Number	Percentage	Number	Percentage
Ratio ≈0	16	1.00%	19	0.74%
0<Ratio<0.90	26	1.63%	25	0.97%
0.90<Ratio<1	18	1.13%	16	0.62%
Ratio = 1	1538	96.25%	2516	97.67%

We found there are some gaps in C4GEM when checking the xylose pathway in the two networks. In AraGEM, there are two pathways to produce xylose, so knockout of UDP-glucose 6-dehydrogenase (UDPGDH, EC:1.1.1.22) will not influence on the biomass synthesis. But in C4GEM, only UDPGDH was responsible for xylose production, the other alternative pathway does not work because of two missing enzymes, xylose isomerase (EC: 5.3.1.5) and xylulokinase (EC:2.7.1.17). We searched the GeneBank database [30] to find that genes (GeneID: 100194128, 100194385) encoding xylose isomerase and genes (GeneID:100282641, 100382670) encoding xylulokinase. So we complemented the xylose pathway in C4GEM, thus the biased results can be avoided.

Next we investigated the effects of particular key enzymes on photosynthesis and biomass synthesis in C3 and C4 plants. Table 5 illustrated these enzymes, their functions and the ratio of objective flux after deletion. '0' means the knocked-out enzyme resulting no flux of biomass or CO_2 _fixation, while '1' means there is no influence on maximal flux of biomass or CO_2 _fixation. Knockouts of enzymes in Calvin cycle have lethal effects on both C3 and C4 networks. For example, the central enzyme of Calvin cycle, Rubisco (EC: 4.1.1.39) catalyzes the fixation of both CO_2 _and O_2_. Its deletion results in zero flux of CO_2 _fixation and biomass, which accords with the fact that photosynthesis and plant growth is positively correlated with Rubisco activity [31,32]. When deleting transaldolase (TAL, EC: 2.2.1.2) in pentose phosphate pathway and glycolate oxidase (LOX, EC: 1.1.3.15) in glyoxylate and dicarboxylate metabolism pathway, the CO2 fixation and biomass will also reduce to zero flux in these two plants [33,34]. Aconitases (EC: 4.2.1.3) is an important enzyme in TCA cycle, its knockout reduced the flux of CO_2 _fixation, and completely no flux of biomass in both C3 and C4 networks [35].

**Table 5:** The effects of key enzyme knockouts on optimal flux of biomass and CO2 fixation

Enzyme	EC	Pathway	Ratio of biomass		Ratio of CO_2 _fixation	
	
			C3	C4	C3	C4
Rubisco	4.1.1.39	Calvin cycle	0	0	0	0
RPI	5.3.1.6	Calvin cycle	0	0	0	0
Prk	2.7.1.19	Calvin cycle	0	0	0	0
RPE	5.1.3.1	Calvin cycle	0	0	0	0
TKT	2.2.1.1	Calvin cycle	0	0	0	0
TAL	2.2.1.2	Pentose phosphate pathway	0	0	0	0
LOX	1.1.3.15	Glyoxylate and dicarboxylate metabolism	0	0	0	0
Aconitases	4.2.1.3	TCA cycle	0	0	0.89	0.82
PGLP	3.1.3.18	Photorespiratory	1	1	1	1
SPP	3.1.3.24	Sucrose biosynthesis	1	1	1	1
Amylase isomerase	2.4.1.18	Transitory starch biosynthesis	0	0	1	1
PEPC	4.1.1.31	C4 photosynthesis	1	0	1	1
PPDK	2.7.9.1	C4 photosynthesis	1	0.96	1	0.98

The knockout of hosphoglycolate phosphatase (PGLP, EC: 3.1.3.18) has no effect on the CO_2 _fixation and biomass synthesis, because it catalyzes the first reaction of the photorespiratory C2 cycle [36]. Sucrose-6(F)-phosphate phosphohydrolase (SPP, EC: 3.1.3.24) catalyzes the final step in the pathway of sucrose biosynthesis [37]. Its deletion has no influence, because sucrose synthesis locates in cytosol and has no direct connection with photosynthesis. Amylase isomerase (EC: 2.4.1.18) is responsible for the synthesis of transitory starch in chloroplast, which is the critical reaction for the normal biosynthesis of storage starch, so its deletion has lethal effect on biomass flux for both C3 and C4 plants [38].

In C4 plants, Phosphoenolpyruvate carboxylase (PEPC, EC: 4.1.1.31) notably performs the initial fixation of atmospheric CO_2 _in photosynthesis, which catalyzes the carboxylation of phosphoenolpyruvate (PEP) in a reaction that yields oxaloacetate and inorganic phosphate [39]. Therefore, knockout of PEPC resulted in zero flux of biomass, which validates its crucial role in C4 photosynthesis. Pyruvate phosphate dikinase (PPDK, EC: 2.7.9.1) catalyzes the conversion of the 3-carbon compound pyruvate into phosphoenolpyruvate. Its deletion reduced the flux of CO_2 _fixation and biomass, which is consistent with experiment results that inhibition of PPDK significantly hinders C4 plant growth [40]. In comparison, these two enzymes have no effect on CO_2 _fixation and biomass in C3 network.

### Correlated reaction sets identified by Sampling

There are some reactions co-utilized in precise stoichiometric ratios and exhibit correlated flux in the metabolic network, which called correlated reaction sets. We used the uniform random sampling method to determine dependencies between reactions which can be further used to define modules of reactions [See Methods section]. The simplified model of the C3 network has 494 reactions, 483 metabolites and narrow range on constraints, which can be separated into 65 modules and the largest module consists of 92 reactions. The simplified model of the C4 network has 826 reactions, 806 metabolites and narrow range on constraints, which can be separated into 113 modules and the largest module consists of 169 reactions. There are more correlated reaction sets in C4 than C3 network.

**Figure 3:**
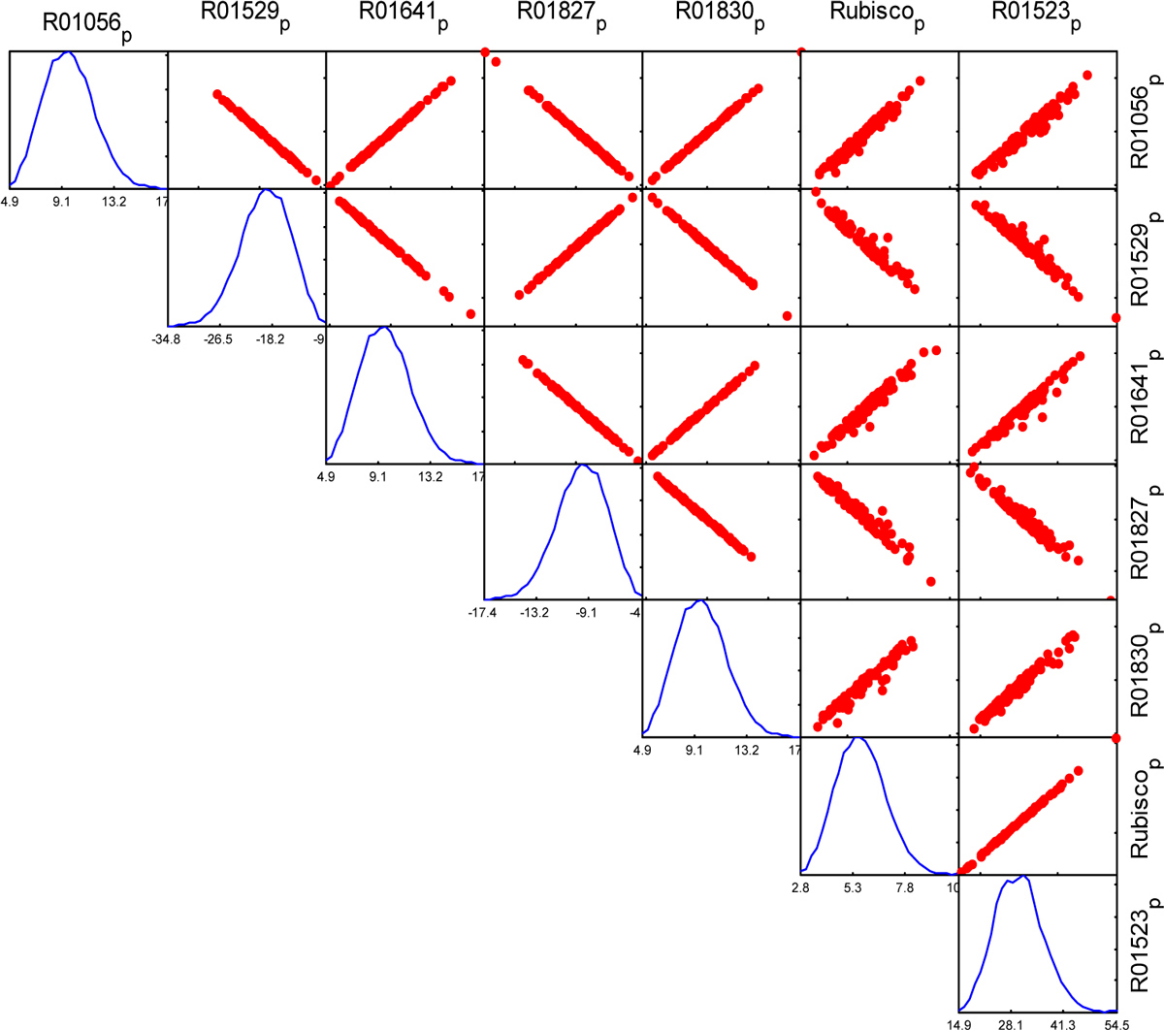
**Correlated reaction sets of Calvin cycle in C3 network**.

**Figure 4:**
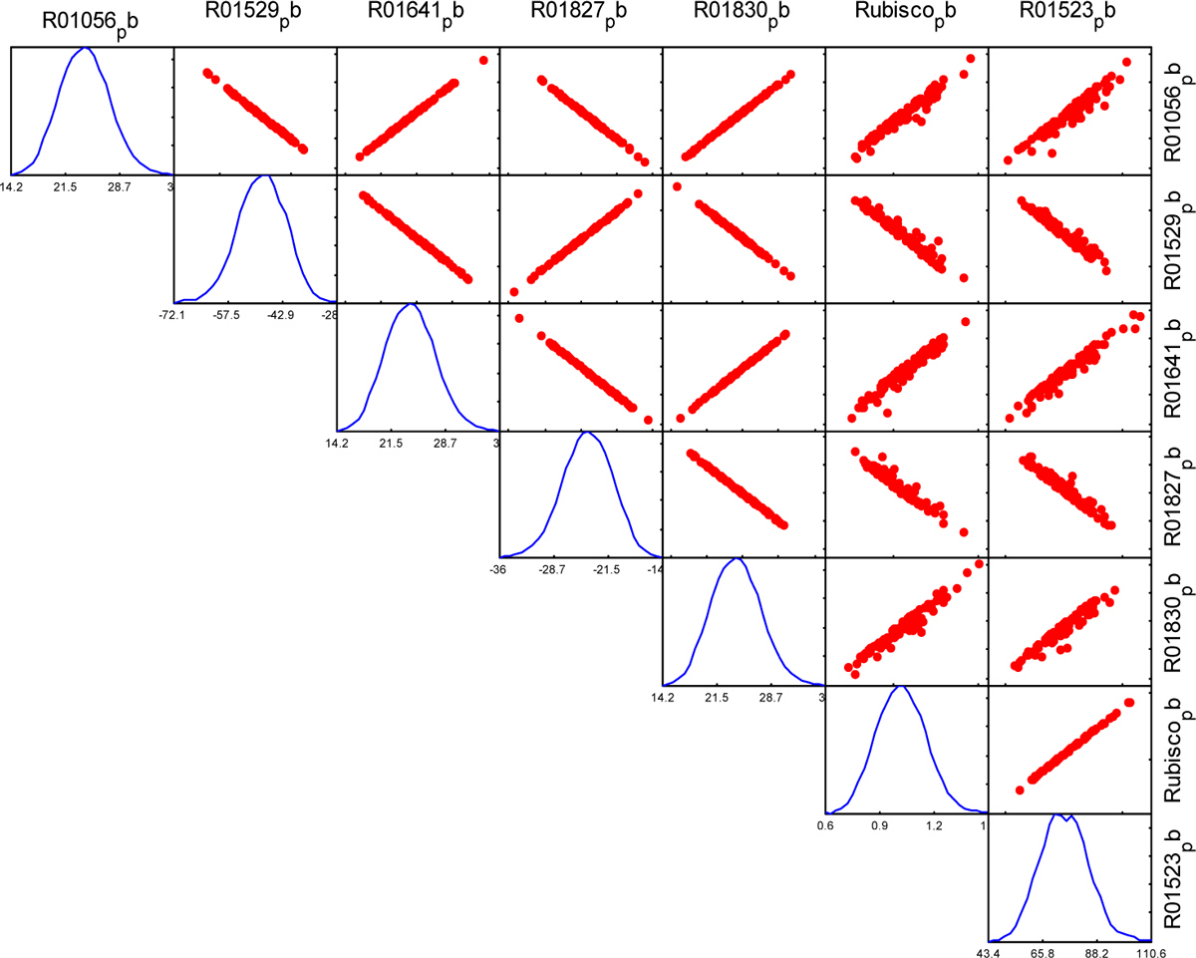
**Correlated reaction sets of Calvin cycle in C4 network**.

**Figure 5:**
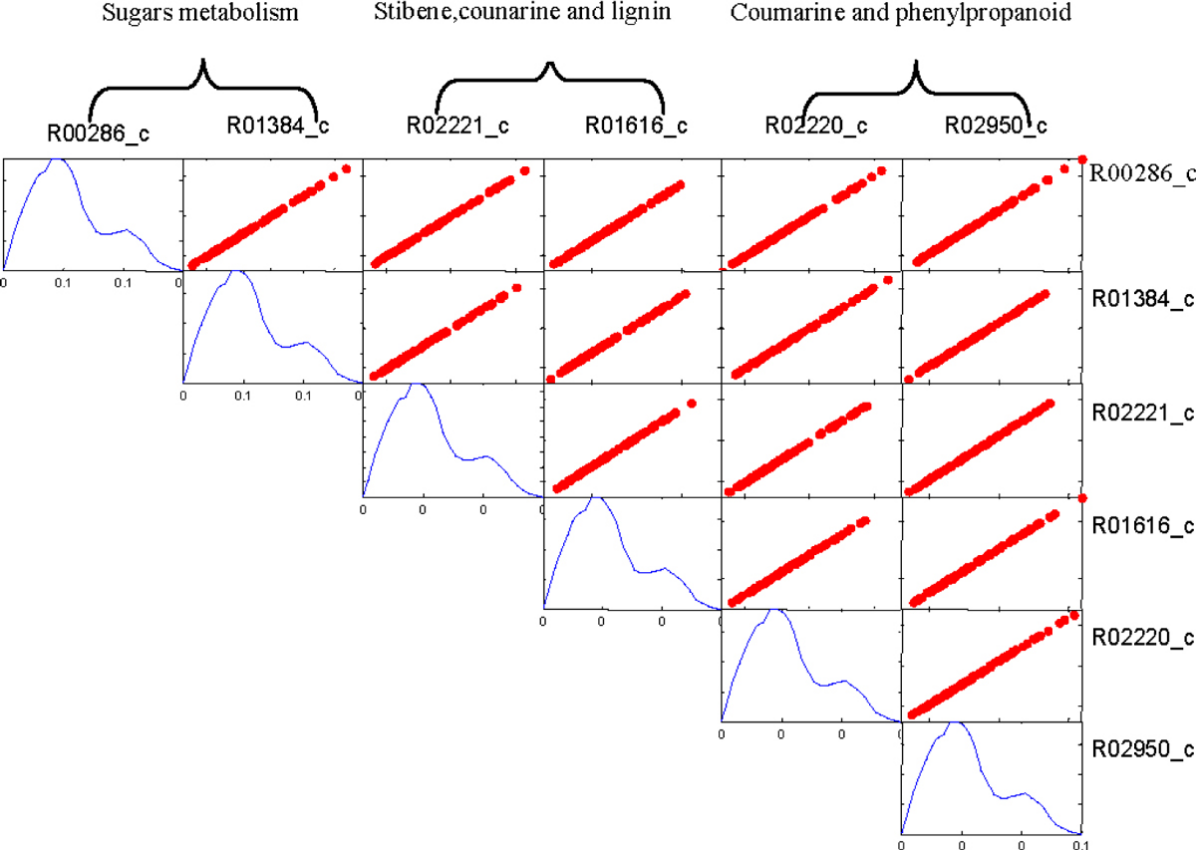
**The reactions from several pathways are correlated in C4 network**.

**Figure 6:**
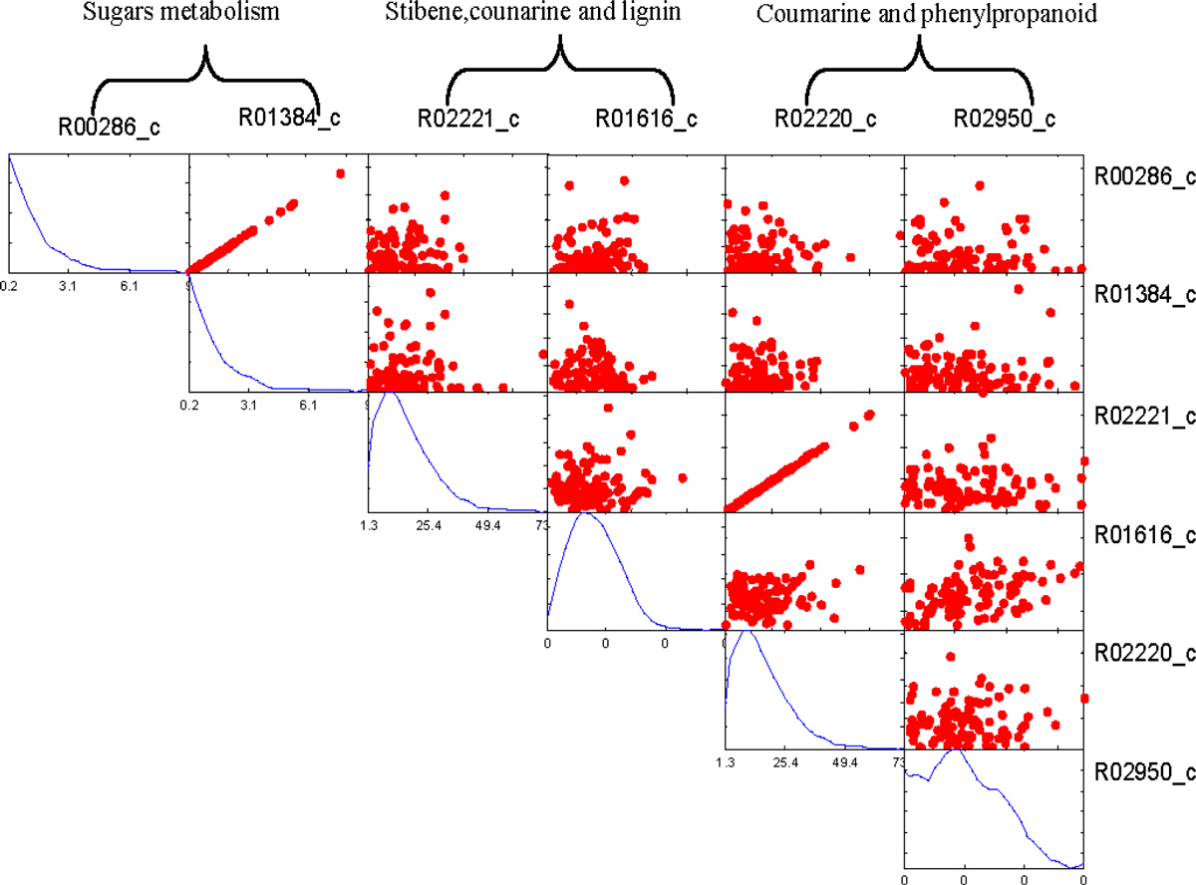
**The reactions from several pathways same with C4 are not correlated in C3 network**.

The fluxes of reactions in the same module exhibit linear correlation. We found the reactions in Calvin cycle are correlated in both C3 and C4 network, as illustrated in Figure 3 and 4 respectively. However, there are some reactions from different pathways also exhibit linear correlation in C4 network, but they are not correlated in C3 model. For example, the reactions from Sugar metabolism, Stibene, counarine and lignin biosynthesis, and Coumarine and phenylpropanoid biosynthesis pathways are significantly correlated in C4 (shown in Figure 5), but no correlation among them in C3 (shown in Figure 6). It demonstrated that C4 plants have better modularity with complex mechanism coordinates the reactions and pathways than that of C3 plants.

### Comparison of response to different environment conditions

**Figure 7:**
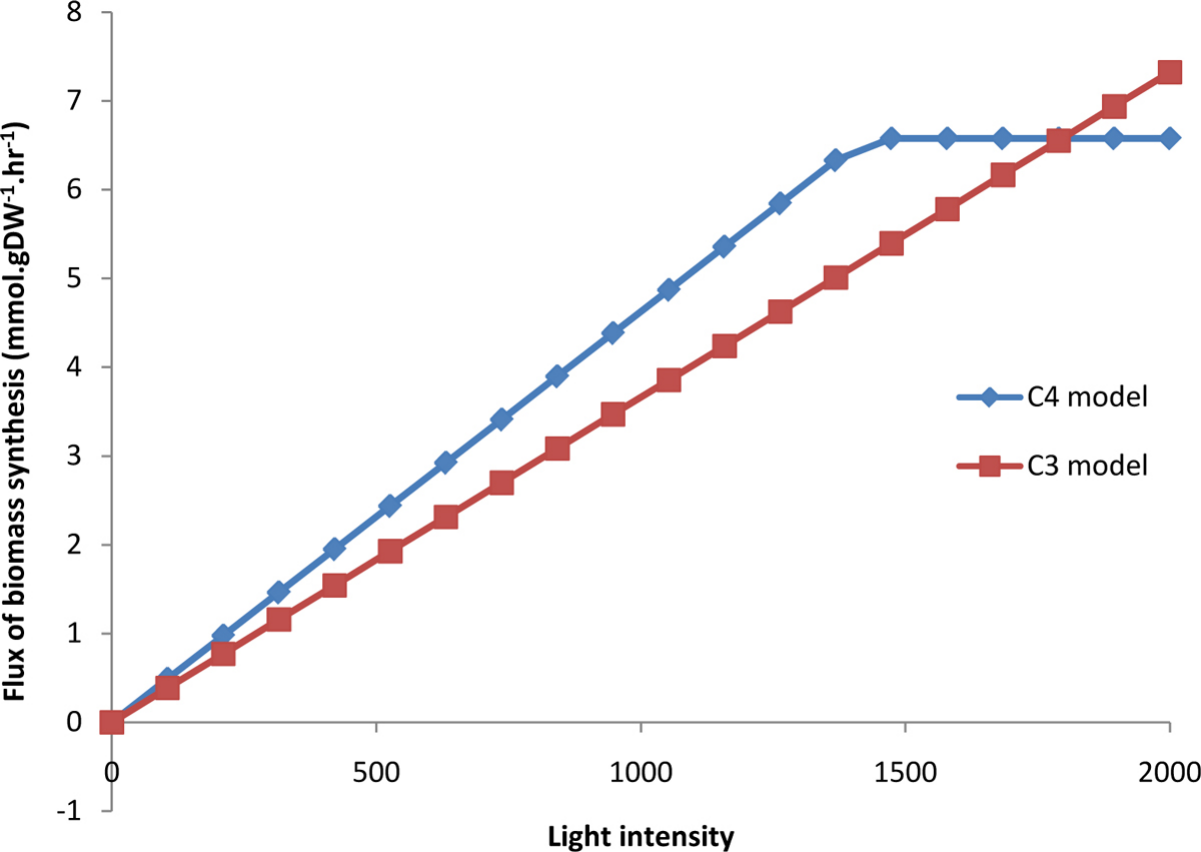
**The effect of light intensity on biomass synthesis in C3 and C4 model**.

**Figure 8:**
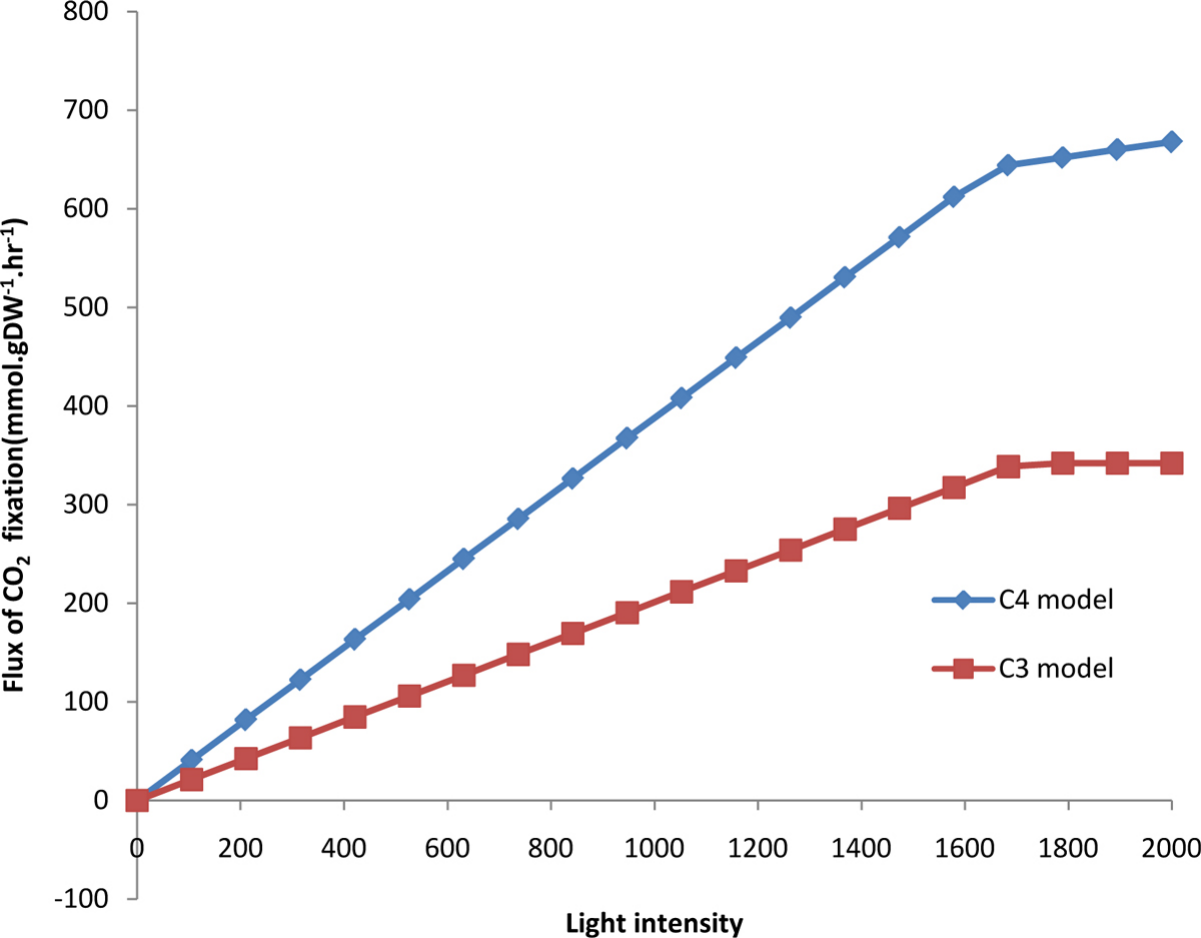
**The effect of light intensity on CO_2 _fixation in C3 and C4 model**.

**Figure 9:**
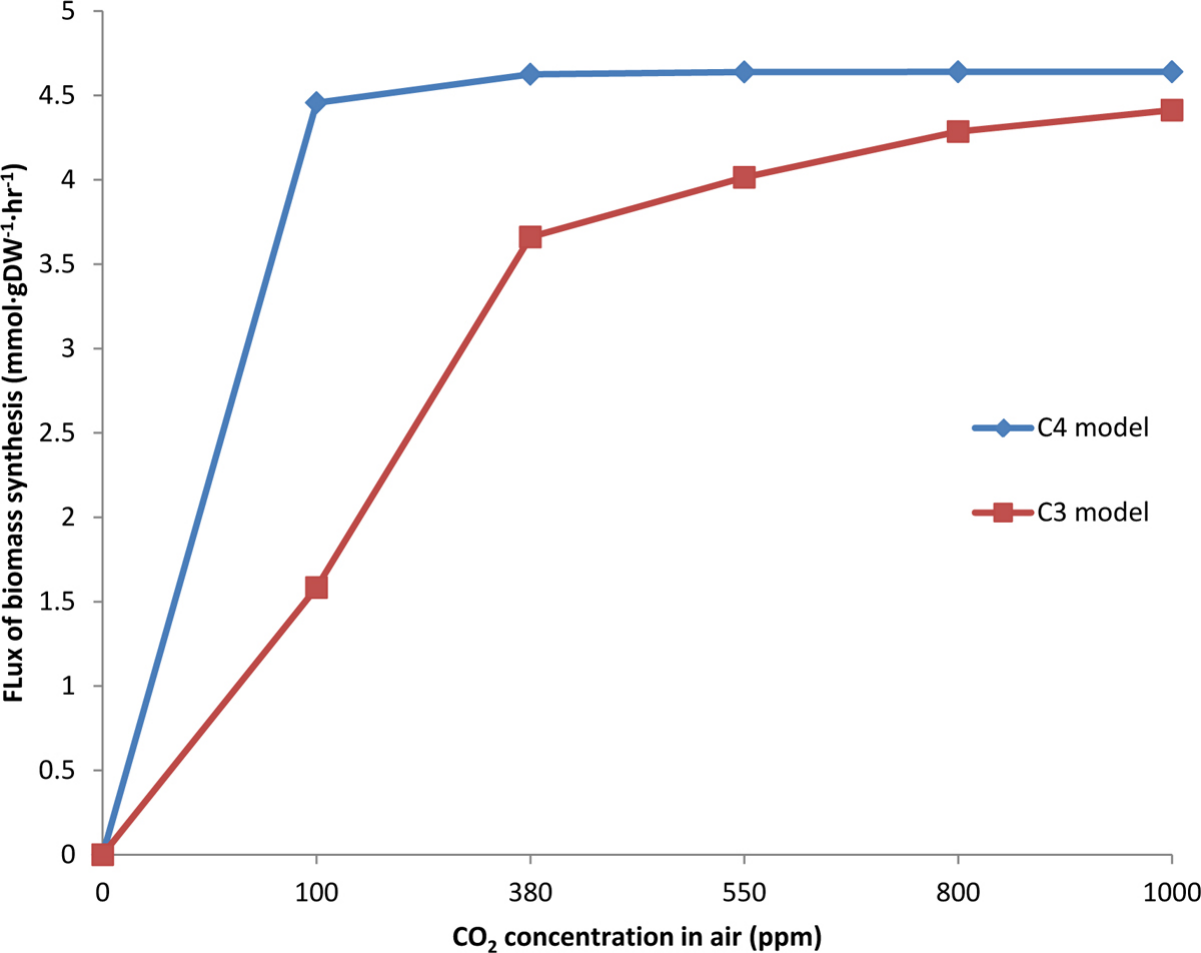
**The effect of CO_2 _concentration on biomass synthesis in C3 and C4 model**.

**Figure 10:**
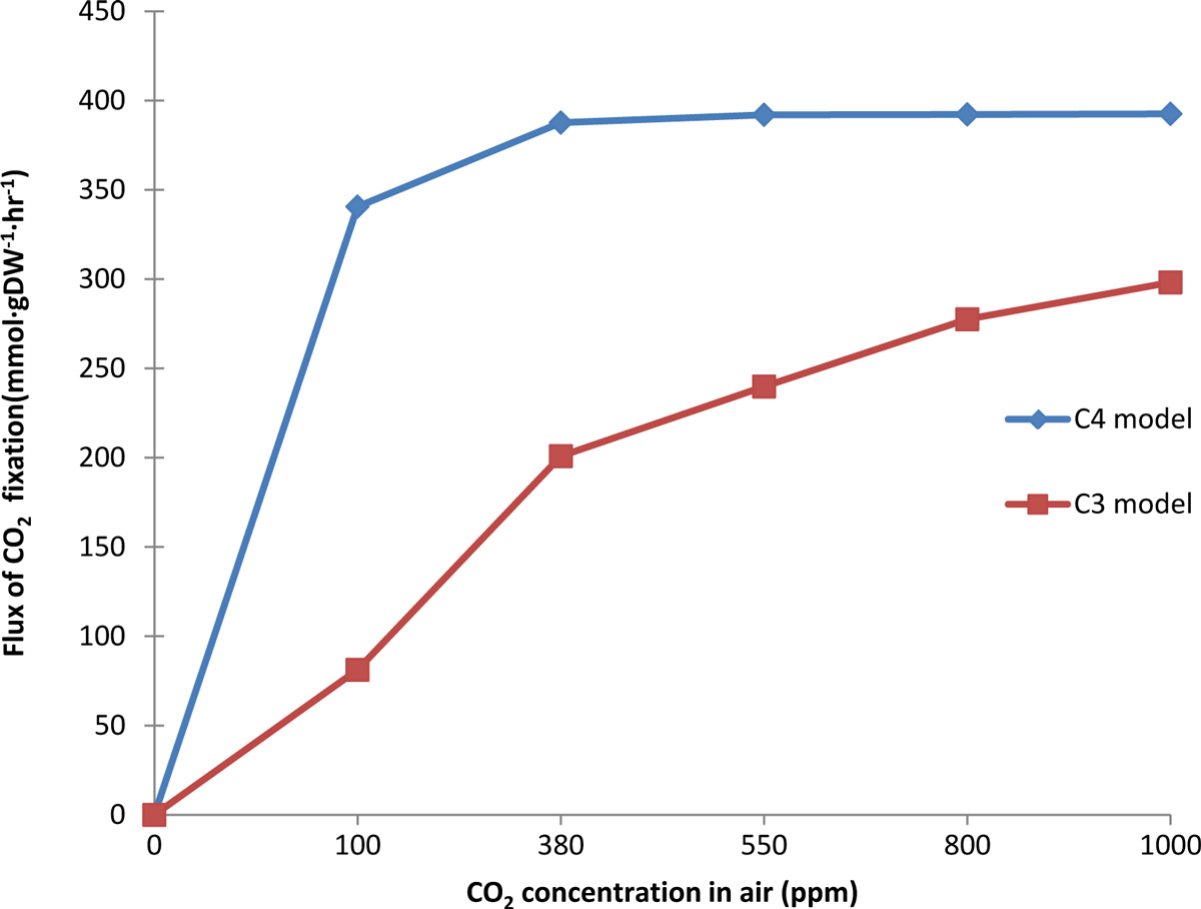
**The effect of CO_2 _concentration on CO_2 _fixation in C3 and C4 model**.

The biomass and CO_2 _fixation of C3 and C4 models were simulated under different light intensity, as shown in Figure 7 and 8. The C3 model (red in Figure 7) and C4 model (blue in Figure 7) presented linear relationship between biomass and light intensity when light intensity is less than 1500. Then with the light intensity increasing, the biomass would be unchanged in C4 model and still increased in C3 model. The C3 model (red in Figure 8) and C4 model (blue in Figure 8) also presented linear relationship between CO_2 _fixation and light intensity when light intensity is less than 1600. Then the CO_2 _fixation was almost keeping unchanged. The increase of both biomass and CO_2 _fixation with light intensity in C4 are faster than that in C3, which reflect more efficient use of solar energy in C4 plants [41]. In addition, we simulated the flux of biomass synthesis and CO_2 _fixation under different CO_2 _concentration, as shown in Figure 9 and 10. The more CO_2 _concentration increases, the more flux of biomass and CO_2 _fixation, and the increase gradually change slowly until to steady state. The simulated curve was consistent with experiment *A-Ci *curve [42]. We found that the increase of both biomass and CO_2 _fixation with CO_2 _concentration in C4 are faster than that in C3, which reflect more efficient use of CO_2 _in C4 plants.

### Contribution of different C4 subtypes to biomass production

C4 plants can be classified to three subtypes according to decarboxylation modes: NADP-malic enzyme (NADP-ME), NAD-malic enzyme (NAD-ME) and PEP carboxykinase (PCK). We explored the influence of each subtype on biomass synthesis and CO2 fixation, by blocking the flux of other two enzymes and giving enough supply of water and nitrogen. As shown in Table 6, for each specific subtype, only the corresponding enzyme has flux and the other two enzymes have zero flux. There are little differences on biomass in the three subtypes. In comparison, the flux of biomass and CO_2 _fixation are maximal in PCK subtype. Moreover, when all the three subtypes are assumed to be active in one metabolism system, the PCK subtype is superior to be used for CO2 decarboxylation. These results are consistent with Fravolini's experiments that photosynthetic performance and above-ground biomass production of B.curtipendula, (PCK subtype) are greater than NADP-ME and NAD-ME types [43]. However, the photosynthesis and biomass of different subtypes also depend on environment conditions, including water and nitrogen supply [44,45]. For example, some species of NADP-ME type show higher rates of photosynthetic and biomass production under low nitrogen availability [46]. Therefore, to clearly elucidate the superiority of C4 subtypes, further design and analysis under multi-factorial combination of environment conditions are required.

**Table 6:** The influences of different C4 subtypes on flux of biomass synthesis and CO_2 _fixation

C4 subtypes	NADP-ME	NAD-ME	PCK	Three Subtypes
**Flux of reactions (mmol·gDW^-1^·hr^-1^)**				
Biomass synthesis	4.52	4.49	4.75	4.90
CO2 fixation	92.20	91.59	96.94	100.01
R00216 (NADP-ME)	79.63	0.00	0.00	0.00
R00214(NAD-ME)	0.00	79.07	0.00	0.00
R00341 (PCK)	0.00	0.00	83.98	86.79

## Conclusions

There is possibility to engineer C4 photosynthesis into C3 plants, because all C4 key enzymes are also present in C3 plants, although the expression levels are much lower than that in C4 species [1]. However it is an enormous challenge. To realize the transition from C3 to C4, systems biology will play a critical role in many aspects, including identification of key regulatory elements controlling development of C4 features and viable routine towards C4 using constraint-based modeling approach [47]. In this study, we improved the current metabolism models AraGEM and C4GEM by setting the ratio of carboxylation and oxygenation by Rubisco, and then systematically compared the constraint-based metabolic networks of C3 and C4 plants for the first time. We found C4 plants have less dense topology, higher robustness, better modularity, and higher CO_2 _and radiation use efficiency, which provide important basis for engineering C4 photosynthesis into C3 plants. In addition, preliminary analysis indicated that the rate of CO2 fixation and biomass production in PCK subtype are superior to NADP-ME and NAD-ME subtypes under enough supply of water and nitrogen. All results are consistent with the actual situation, which indicate that Flux Balance Analysis is a useful method to analyze and compare large-scale metabolism systems of plants.

## Methods

### Determination of the ratio between carboxylation and oxygenation

We improved AraGEM and C4GEM by setting the ratio ofcarboxylation and oxygenation by Rubsico, which has not been conducted in any plant metabolic system. For C3 plants, the ratio *r *between carboxylation and oxygenation under specific CO_2 _and O_2 _concentration can be calculated by the following (4-6).

(4) $$Vco_{2}=\frac{co_{2}}{co_{2}+K_{c}\left(1+\frac{O_{2}}{K_{o}}\right)}$$

(5) $$Vo_{2}=\frac{O_{2}}{O_{2}+K_{o}\left(1+\frac{co_{2}}{K_{c}}\right)}*0.21$$

(6) $$r=\frac{Vco_{2}}{Vo_{2}}$$

Equation (5) and (6) include mechaelis constants for CO_2 _with *K_c _*= 460μbar and O_2 _with *K_o _*= 330mbar [28]. The O_2 _concentration is 210 mbar and the intercellular CO_2 _concentration is about 70 percent of CO_2 _in air, which is 380μbar under standard condition.

Unlike C3 plants, C4 photosynthesis requires the coordinated functioning of mesophyll and bundle sheath cells by CO_2 _concentrating mechanism. The ratio *r *of carboxylation to oxygenation can be expressed as equation (7-15) [48]:

(7) $$Vp=\text{min}\left\{\frac{Cm*Vp\text{max}}{Cm+Kp},Vpr\right\}$$

(8) Ac=min{(Vp+gs*Cm-Rm),(Vcmax-Rd)}

(9) $$Aj=\frac{\left(1-x\right)Jt}{3}-Rd$$

(10) $$A=\text{min}\left\{A_{c},A_{j}\right\}$$

(11) if Ac=Vp+gs*Cm-Rm

(12) $$Cs=\frac{\gamma *Os+Kc\left(1+Os/Ko\right)\left(\left(Ac+Rd\right)/Vc\text{max}\right)}{1-\left(Ac+Rd\right)/Vc\text{max}}$$

(13) $$r=\frac{Vc}{Vo}=\frac{Cs}{2\gamma *Os}$$

(14) else Ac<Vp + gs*Cm-Rm 

(15) $$r=\frac{Vc}{Vo}=\frac{Cs}{2\gamma *Os}=\frac{Cm*gs+Vp-A-Rm}{2\gamma *\left(\frac{\alpha A}{0.047}+Om*gs\right)}$$

Where *C_s _*and *C_m _*are CO_2 _partial pressures respectively in bundle sheath and mesophyll cells; *O_s _*and *O_m _*are O_2 _partial pressures in the two cells; *V_p _*is the rate of PEP carboxylation; *V_pmax _*(120μmol·m^-2^·s^-1^) is the maximum PEP carboxylation rate; *K_p _*(80μbar) is Michaelis constant of PEP carboxylase for CO_2_; *V_pr _*(80μmol·m^-2^·s^-1^)is the constant rate of PEP regeneration; *g_s _*(3mmol·m^-2^·s^-1^) is the physical conductance to CO_2 _leakage; *A_c _*is Rubisco-limited rate of CO_2 _assimilation; *A_j _*is electron-transport-limited rate; *A *is the CO_2 _assimilation rate; *V_cmax _*(60μmol·m^-2^·s^-1^) is the maximum Rubisco activity; *γ *(0.5/2590) is half the reciprocal of Rubisco specificity; *R_d _*= 0.01*V_cmax _*= 0.6μmol·m^-2^·s^-1 ^is leaf mitochondrial respiration; *R_m _*= 0.5 *R_d _*= 0.3μmol·m^-2^·s^-1 ^is mesophyll mitochondrial respiration; *α *(0<*α*<1, *α *were assumed to be zero in our results) is fraction of PSII activity in the bundle sheath; *x *(*x *= 0.4) is partitioning factor of electron transport rate. *J_max _*(400μmol electron m^-2^·s^-1^) is maximal electron transport rate; *K_c _*(650μbar) for CO_2 _and *K_o _*(450mbar) for O_2 _are mechaelis constants of Rubisco. In C4 plants, CO_2 _concentration in mesophyll cell is only 37 percent of CO_2 _in air [49] and the other parameters can be obtained in [48].

### Topological parameters in metabolic network

The topological properties of metabolic network can be analyzed based on graph theory, which can reflect the structure and robustness of large-scale network. In this study, the reactions are represented as nodes, if the product of reaction A is the substrate of a reaction B, there will be an edge from A to B. We consider some important parameters including degree, clustering coefficient, betweenness centrality and distance (path length). The degree of a node is the number of edges connected with other reactions. Degree centralization of a network is the variation in the degrees of vertices divided by the maximum degree variation which is possible in a network of the same size. Clustering coefficient is used to compute different inherent tendency coefficients in undirected network. Betweenness centralization is the variation in the betweenness centrality of vertices divided by the maximum variation in betweenness centrality possible in a network of the same size. The distance between two nodes is the shortest path length from one to the other. The diameter of network is the maximal distance among all pairs of nodes. All the topology analysis was conducted using the visual software Pajek [26].

### Flux Balance Analysis

The biochemical reactions can be represented mathematically in the form of a stoichiometric matrix *S*, the flux through all reactions in a network is represented by the vector *v*, so the system of mass balance equation at steady state is given as *Sv = 0*. In any realistic large-scale metabolic model, there are more reactions than compounds, so there is no unique solution to this system of equations. Flux Balance Analysis (FBA) can solve the flux distribution by setting a set of upper and lower bounds on *v *and optimizing some objective function with linear programming, as following:

$$\begin{array}{c} \text{Maximize or minimize}\;\text{Z}={\text{c}}^{\text{T}}\text{v}\\ \;\;\text{subjectto}\;\text{Sv}=0\\ \;\text{and}\;{\text{v}}_{\text{m i n}}\leq \text{v}\leq {\text{v}}_{\text{m}} \end{array}$$

Where *c *is a vector of weights indicating how much each reaction contributes to the objective function. In this study, we choose CO_2 _fixation and biomass synthesis as two objective functions.

The COBRA toolbox is a free MATLAB toolbox for performing the simulation. The fluxes that are identified at various perturbations can be compared with each other and with experimental data.

### Uniform random sampling

Uniform random sampling of the solution space in any environmental condition is a rapid and scalable way to characterize the structure of the allowed space of metabolic fluxes. Before the sampling was performed, the effective constraints for each reaction were calculated using the method of Flux Balance Analysis in COBRA toolbox [50]. Specifically in sampling, COBRA toolbox uses an implementation of the artificial centered hit-and-run (ACHR) sampler algorithm with slight modifications to generate such a set of flux distributions that uniformly sample the space of all feasible fluxes. Initially, a set of 5000 non-uniform pseudo-random points, called warm-up points, was generated. In a series of iterations, each point was randomly moved while keeping it within the feasible flux space. This was accomplished by choosing a random direction, computing the limits on how far a point could travel in that direction (positive or negative), and then choosing a new point randomly along that line. After numerous iterations, the set of points was mixed and approached a uniform sample of the solution space [51] and 2000 points was loaded for analysis. The sampling procedure can be achieved with the function 'sampleCbModel' and the correlated reaction sets can be identified by 'identifyCorrelSets' in the COBRA toolbox. Correlated reaction sets are mathematically defined as modules in biochemical reaction network which facilitate the study of biological processes by decomposing complex reaction networks into conceptually simple units. This sampling approach is used to fully determine the range of possible distributions of steady-state fluxes allowed in the network under defined physicochemical constraints and used to analyze the general properties of networks by testing their robustness to parameter variation [50].

## Competing interests

The authors declare that they have no competing interests.

## Authors' contributions

ZW designed the project and analysis methods. CLW conducted the analysis of metabolic network topology and flux distribution. LYG improved the model by setting the ratio between carboxylation and oxygenation by Rubisco. YXL managed the project. CLW and ZW wrote the manuscript.

## Supplementary Material

Additional file 1 The constraints in the improved models of C3 and C4 metabolic networks Click here for file
